# The Case of the Neurasthenic

**Published:** 1919-06-28

**Authors:** 


### DANGERS OF INSTITUTIONAL TREATMENT.

### The Case of the Neurasthenic.

It has long been more or less recognised that curative treatment at residential institutions is liable to cure the body at the expense of the character. The life, for instance, led at sanatoria for tuberculosis is frequently found to produce introspection and indolence. When every detail is arranged for the patient, when he is encouraged to lead a lazy life both physically and mentally, when his will is subject to the powers that be, and when his main interests are his food and his temperature, a certain flaccidity of morale and a "fushionlessness" of character are very likely to ensue. He loses taste for work: he becomes a lounger and a loiterer on the ways of life. This is especially the case where treatment is prolonged and where the patient has no natural mental initiative or intellectual tastes or æsthetic interests.

The war has undoubtedly accentuated these institutional dangers. In the first place it has produced in the public, who are prone to hero worship, a natural inclination to pet and pamper the patient, and in the patient, who has been exposed to hardships and dangers, a desire to be pampered and petted. An appreciation of the "cushy" is inevitable after a Spartan existence in a trench; and a man who has had rough army fare for months takes very kindly to chicken-breast and champagne. That is very natural and up to a certain point harmless enough; but if the coddling be too prolonged it undoubtedly leads to a paralysis of the will, to a slackening of moral and mental fibre, to a loss of ambition. When a man has perforce lived a sheltered, idle life for twelve or twenty months, when he has been waited on hand and foot by indulgent nurses, and has had theatres and cinemas, and motor-drives to give colour to his days, is it any wonder if he sings with the lotus-eaters?—

> "We have had enough of action . . . .
> Surely, surely slumber is more sweet than toil, the shore
> Than labour in the deep mid-ocean, wind and wave and oar;
> O rest ye, brother mariners, we will not wander more."

The danger of such degeneration is especially present in the case of neurasthenics. Neurasthenia is often a vague, indefinite complaint with symptoms mainly subjective. From its very nature it involves protracted rest, implies volitional as well as nervous asthenia, and easily relapses into indolence, valetudinarianism, with unconscious malingering. When the neurasthenic—as is often the case—has been treated at various general hospitals, before reaching a hospital specially for nerve cases, the chances are that injudicious sympathy has already produced a condition of moral flaccidity and of hypochondria that render cure of the purely neurasthenic symptoms doubly difficult. Suggestion has sown its seeds; indeed, the very word "neurasthenia" has itself suggested many of the symptoms. Suggestion may not be so potent in the uncomplicated neurasthenic case, as in a case of hysteria, but it is often more difficult to dispel its effects.

There can be no doubt that many of the chronic cases of neurasthenia, passing from institution to institution, have a large factor of suggestion and habit in them, and require nothing so much as a motive and spur to make them work—a motive and spur often completely removed by a pension and the philanthropy of kind friends.

Though lack of self-confidence and lack of energy may be quite genuine, they are not unfrequently merely the residual result of prolonged institutional shelter and institutional coddling. A man who has not used his brains or his will-power for many months must expect to be some time in recovering full use of them, and neither self-confidence nor energy are likely to return unless the patient re-educate his disused faculties and exercise his atrophied will. The writer, who has had personal experience of neurasthenic cases, urges that in almost all cases neurasthenics should be given definite work to do during treatment. In this connection it may be mentioned that the Kitchener House at Regent's Park and the Kitchener House at Hampstead are both doing excellent work in providing occupation and training for invalid soldiers. Often the neurasthenic will never be fit to return to his pre-war occupation, and advantage may be taken of his freedom from harness to train him in some new trade; but, again, patients are tempted to exploit their condition and to make it an excuse for declining an old job with a view to finding a "cushier" one. We have known of cases too neurasthenic to do ordinary draughtsman's work, yet able to undergo a musical training requiring a much greater expenditure of nerve energy; and a case too neurasthenic to be a clerk, yet quite ready to go on tour with a theatrical company. Pains must be taken to find suitable work for neurasthenics, but they must not be allowed to drift about, and must not be allowed to exploit their weakness.

The neurasthenic must be seriously considered as a social problem, and we do not think it is an exaggeration to say that mismanagement of neurasthenics will have even political repercussions. The neurasthenic, especially the neurasthenic with a mental element, or with an element of conscious or unconscious malingering in his case, is apt to be suspicious, anti-social, and emotional. He is quick, too, to find or to invent grievances, and at this time of labour unrest the unemployed neurasthenic will almost certainly become a centre of discontent and disorder.

For all which reasons we urge that the dangers of prolonged institutional treatment should be realised, and that efforts should be made to cure not only the bodies of the soldiers, but to send these men back to society willing and ready to work, and with some sense of their social duties and responsibilities.

